# Assessment of the risks of a myasthenic crisis after thymectomy in patients with myasthenia gravis: a systematic review and meta-analysis of 25 studies

**DOI:** 10.1186/s13019-020-01320-x

**Published:** 2020-09-29

**Authors:** Chaoying Liu, Peng Liu, Xiao jing Zhang, Wen qian Li, Guoyan Qi

**Affiliations:** Hebei Key Laboratory of Myasthenia Gravis, Center of Treatment of Myasthenia Gravis Hebei Province, First Hospital of Shijiazhuang, No. 9 Fangbei Road, Chang’an District, Shijiazhuang, 050011 Hebei Province China

**Keywords:** Myasthenic crisis, Thymectomy, Myasthenia gravis, Meta-analysis, Risks

## Abstract

**Background:**

Despite the burgeoning literature describing preoperative and postoperative risks of a myasthenic crisis after thymectomy (MCAT) in patients with myasthenia gravis, substantial differences exist in the risk factors identified by previous studies. We conducted a meta-analysis to assess the reported risk factors and MCAT risk.

**Methods:**

We collected relevant studies on the risk factors for MCAT by searching the PubMed, Embase, The Cochrane Library, China Biology Medicine (CBM), WanFang Data, VIP and CNKI databases. The search period ranged from the establishment of the database to November 2019.

**Results:**

Twenty-five of the 458 identified studies were eligible for the meta-analysis. Seven retrospective cohort studies and 18 case-control studies were included, and 14 risk factors for MCAT were extracted. **Meta-analyses of the association between MCAT and risk factors related to the patient’s preoperative condition included a** preoperative history of MC, preoperative bulbar symptoms, IIa + IIb + III + VI, IIb + III + VI, VI + V, dosage of pyridostigmine bromide prior to the operation, a preoperative AchR-Ab level > 100 (nm/L), preoperative pulmonary function, preoperative complications, and preoperative disease course. **Meta-analyses of the association between MCAT and surgery-related risk factors included** intraoperative blood loss > 1000 mL and the mode of operation. **Meta-analyses of the association between MCAT and postoperative risk factors included** postoperative lung infection, thymoma and the WHO classification. The operation time was not an independent risk factor for MCAT.

**Conclusions:**

The independent risk factors for MCAT were a preoperative history of MC, preoperative bulbar symptoms, preoperative MG Osserman stage, preoperative dosage of pyridostigmine bromide, preoperative serum AchR-Ab level, lung function, major postoperative complications, disease duration before thymectomy, blood loss, thoracotomy, postoperative lung infection, thymoma, and WHO classification.

## Introduction

Myasthenia gravis (MG) is an autoimmune disorder induced by neurotransmission defects at the neuromuscular junction and is characterized by muscle weakness and fatigue [1]. MG is typically treated with anticholinesterase agents, surgical thymectomy, or immunosuppression and with short-term immunotherapies (plasmapheresis and intravenous immunoglobulin administration) to reduce the risk of exacerbations [2]. Thymectomy has been selected as a surgical treatment for patients with MG since Blalock reported its efficacy as a treatment for MG complicated with or without thymoma [3, 4]. However, 3–30% of patients with MG still develop a myasthenic crisis after thymectomy (MCAT) [5–8]. An MG crisis (MC) represents a serious, life-threatening, rapid worsening of MG and potential airway compromise due to ventilatory or bulbar dysfunction [9]. MC is the main cause of death after MG thymectomy. Many risk factors for MCAT have been reported, including internal factors and surgical factors, such as a myasthenic crisis, medulla oblongata muscle weakness and operation time. Geng et al. [10] performed a meta-analysis of risk factors for a myasthenic crisis after thymectomy that included 15 trials and 2626 patients with MG. However, substantial differences exist in the risk factors for MCAT identified by previous studies. The included studies exhibited substantial heterogeneity, and the previous meta-analysis [10] did not provide any details in their methods section on their plan to address the heterogeneity. The aetiology of MCAT remains unclear, and an effective risk assessment system for predicting the occurrence of MCAT is not available. Therefore, we conducted a meta-analysis to assess reported risk factors and the risk of MCAT and to achieve an adequate sample size required for a comprehensive and precise estimation of the associations between these factors and MCAT.

## Methods

### Literature sources and search strategy

We collected relevant studies examining the risk factors for MCAT by searching the PubMed, Embase, The Cochrane Library, CBM, WanFang Data, VIP and CNKI databases. The search period extended from the establishment of the database to November 2019.

The search was conducted using a combination of subject words and free words. We used the following search terms: (myasthenic crisis) AND (“thymectomy [MeSH Terms]” OR “thymectomies”).

We also used combinations of the following search terms to expand the search: (myasthenic crisis) AND (“risk factors [MeSH Terms]” OR “factor*, risk” OR “risk factor” OR “population* at risk” OR “risk, population* at”).

### Inclusion criteria

The following inclusion criteria were used: (1) Patients with MG after thymectomy; (2) Intervention: provides risk factors for patients with MG after thymectomy; (3) Comparator: division of participants into a case group and a control group according to the occurrence of a myasthenic crisis; (4) Outcome: risk of MCAT reported as an adjusted odds ratio (OR) and corresponding 95% confidence interval (CI); and (5) Study design: observational studies including cohort, case-control, or cross-sectional studies. No language restrictions were imposed.

Exclusion criteria included studies, animal experiments, case reports, reviews, expert opinions and editorials that did not provide sufficient data for analysis.

### Data extraction and quality assessment

Two researchers (Li WQ and Zhang XJ) independently screened the literature, extracted data and evaluated the risk of bias of the included studies. If they had a difference of opinion, they would reach an agreement through a consultation or by discussing the matter with a third person. In our study, we used a homemade data extraction table to extract data, including the study source; number of years; type, location, and number of patients; number of patients with MC; age at the operation; age of onset; and significant risk factors and results.

This systematic review was based on a meta-analysis of epidemiological observational studies (MOOSE) [11]. We used the Newcastle-Ottawa Scale (NOS) to assess the methodological quality, risks of selection and cohort comparability bias, and outcomes of the included studies [12]. The score of this scale ranges from 0 to 9 points. Studies with NOS scores of 5 or more stars were considered high quality; otherwise, the quality of the study was considered poor, and these studies were excluded.

### Data analysis

RevMan 5.3 statistical software and Stata 15.0 software were used for statistical analysis. The same potential predictors must have been studied at least twice in the analysis of the original data. For dichotomous results, the adjusted OR of the 95% CI was calculated. For continuous results, the mean difference (MD) of the 95% CI was calculated.

We used Cochrane’s Q statistics and I^2^ statistics to study heterogeneity. Heterogeneity was classified as low, medium, or high based on I^2^ values of 25, 50%, or 75%, respectively [13]. If *P* > 0.1 and I^2^ ≤ 50%, homogeneity was present and a fixed effect model was adopted; *P* ≤ 0.1 and I^2^ > 50% indicated the presence of heterogeneity. We subsequently conducted a sensitivity analysis or subgroup analysis to explore possible explanations for the heterogeneity. For the predictors whose source of heterogeneity was not identified in the sensitivity or subgroup analysis, a random effect model was used in the meta-analysis. Publication bias was examined using Begg’s test, with a *P* value < 0.1 indicating a significant difference.

## Results

### Search results

**Fig. 1:**
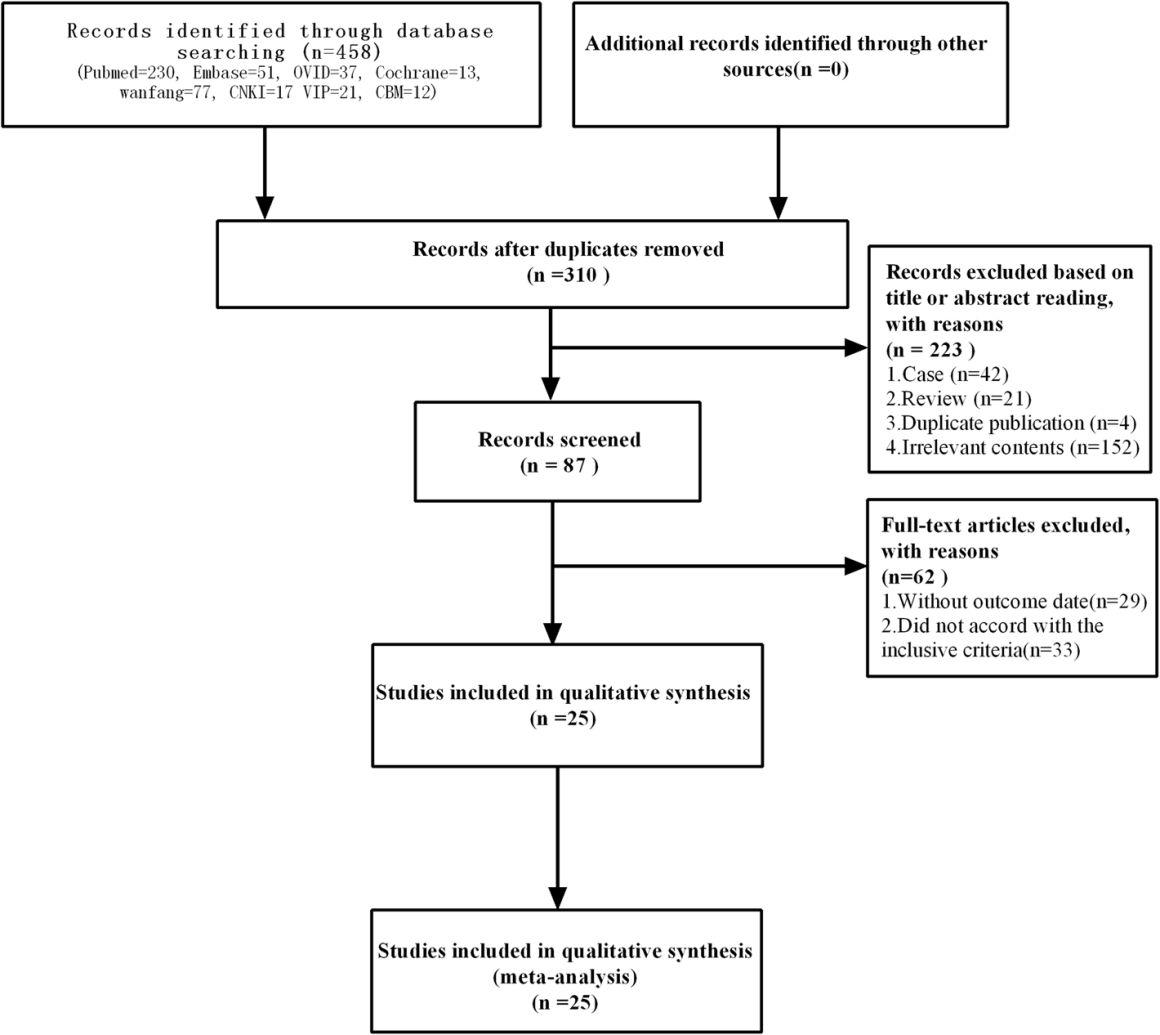
Flow diagram of the literature search and selection process

Four hundred fifty-eight articles were retrieved. First, 148 duplicate records were excluded. Second, 223 records were excluded after reading the titles and abstracts of the articles. Then, 62 records were excluded by reading the full texts. Finally, data from 3728 individual patients with 692 myasthenic crisis cases were included in the meta-analysis of 25 eligible studies [1, 7, 8, 14–36]. Figure 1 presents the literature search and study selection process.

### Basic characteristics of the included studies and risk of bias assessment

Seven retrospective cohort studies and 18 case-control studies were included, and 14 risk factors for MCAT were extracted. The basic characteristics of the included studies are shown in Table 1. The NOS criteria used to evaluate the quality of the 25 studies that were included in this meta-analysis are shown in Table 1 and Table 1 in the supplement. Significant risk factors associated with MCAT and the results of bias risk assessment are shown in Table 2.

**Table 1:** The basic characteristics of the included study

Source	Year	Study year	Study design	No. of patients	No. of myasthenic crisis cases	Age at thymectomy (mean) Age, years	Gender Male/women	Study region	Non-thymomatous/thymoma	Significant risk factors	NOS score
Kato	2019	2000–2015	retrospective cohort study	90	14	median (range) 51.5 (41.3–64.0)	41/46	Japan	33/57	(15)	8
Xue	2017	2005–2014	case-control studies	127	13	NA	68/59	china	127	(3),(10)	8
Zou	2016	2007.06–2013.12	retrospective cohort study	541	67	26.4 ± 11.7/27.06 ± 13.5	256/285	china	NA	(1),(4),(6),(16)	6
Ando	2015	2000.01–2013.12	case-control studies	55	10	median (range) 55 (13–79)	25/30	Japan	30/25	(1),(17)	7
Lee	2015	2007.10–2012.03	case-control studies	146	10	T/C 25.0 (15.5–48.0)/35.0 (27.0–46.0)	NA	Korea	105/41	(12),(18)	8
Li	2018	2000.01–2013.03	case-control studies	176	51	46.6 ± 11.7	90/83	China	0/173	(2),(19)	9
Yu	2014	1997.03–2012.03	retrospective cohort study	178	58	38.4 ± 13.2	95/83	China	69/109	(1),(2),(8)	8
Choi	2014	1996.01–2009.12	retrospective cohort study	49	12	50.3 ± 12.4	23/25	Korea	0/49	(12)	6
Leuzzi	2014	1995.01–2011.12	case-control studies	177	22	45.8 ± 16.8	107/70	Italy	53/124	(1),(3),(4),(14)	9
Nam	2011	1997.01–2007.12	retrospective cohort study	68	20	age at onset 43.6 ± 13.9	NA	Japan	28/38	(1)	9
Chu	2011	1990–2009	case-control studies	243	44	31.5 ± 15.7	124/119	China	175/68	(3),(5),(13)	5
Liu	2006	1990.01–2006.01	case-control studies	176	36	31 ± 14	74/102	China	122/54	(1),(2),(6),(24)	9
Li	2016	1970.05–2011.05	case-control studies	306	49	31.54 ± 16.37	155/151	China	NA	(1),(2),(5)	8
Qian	2016	2002.02–2015.06	case-control studies	86	16	median (range)47 (25–71)	37/49	China	40/46	(1),(2),(3)	8
Li	2017	2008.01 ~ 2015.01	case-control studies	63	12	median (range)37 (10–76)	25/38	China	41/22	(3),(8),(9),(10)	7
Liu	2014	2011.01–2014.02	case-control studies	102	42	T/C 46.7 ± 10.11/39.6 ± 6.41	45/57	China	36/66	(3),(5),(11),(12),(20)	6
Niu	2013	2002.07–2012.07	case-control studies	134	28	age at onset 35 (1–82)	66/68	China	69/65	(3)	6
Ma	2011	1995.07–2009.12	case-control studies	84	24	median (range)42.67 (16—68)	35/49	China	21/63	(1),(3),(8),(11)	6
Zhang	2015	2008.06–2014.06	case-control studies	58	15	T/C 60.60 ± 6.20/60.7 ± 6.3	26/32	China	0/58	(3),(11),(21)	6
Wang	2006	NA	case-control studies	126	13	median (range)38 (9–62)	54/72	China	56/70	(1),(3),(7),(22)	5
Chen	2007	2002.07–2005.12	case-control studies	101	29	33.2 ± 14.67	43/58	China	33/68	(1),(3),(4),(13),(23)	8
Ge	2019	2008.01–2018.01	retrospective cohort study	47	14	40.1 ± 16.7	22/25	China	24/23	(11),(13)	6
Li	2014	2008.01–2013.06	case-control studies	198	32	NA	NA	China	135/63	(1),(3),(5),(6)	5
Kanai	2017	2002.01–2014.12	case-control studies	275	17	T/C:45.8 ± 16.1/49.2 ± 15.5	106/169	Japan	145/130	(2),(12),(14)	9
Watanabe	2004	1985.01–2002.12	retrospective cohort study	122	44	44 ± 17	30/92	Japan	93/29	(2),(7),(9)	6

*MG* myasthenia gravis;*AchR-Ab* anti-acetylcholine receptor antibody; *POA* preoperative anxiety

(1) preoperative MC history (2) preoperative bulbar symptom (3) preoperative Osserman stages (4) postoperative lung infection (5) thymoma (6) preoperative dosage of pyridostigmine bromide (7) preoperative serum AchR-Ab level (nm/L) (8) operation time (9) intraoperative blood loss>1000 ml (10) WHO classification (11) mode of operation (12) preoperative lung function (13) major postoperative complications (14) disease duration before thymectomy (15) Masaoka stage (16) POA (17) unstable MG (18) decremental response of orbicularis oculi (19) incomplete resection (20) general anaesthesia (21) Postoperative medication (22) Potential MC (23) age (24) preoperative lung infection

**Table 2:** Significant risk factors associated with MCAT and the results of bias risk assessment

Significant Risk Factors		No. of studies	No. of MC	No. of total patients	Analysis model	Adjusted OR (95%CI)	Study heterogeneity			Test for overall effect		Begg’s test P value
							**Q**	**P**	**I**^**2**^**,%**	**Z**	***P*** **value**	
**Preoperative patient condition-related risk factors**												
Preoperative MC history		12	377	2094	Fixed	4.13 [3.08, 5.54]	13.46	0.26	18	9.46	<0.00001	0.034
	without	ref										
	with											
Preoperative bulbar symptoms		8	254	1443	Fixed	3.71 [2.54, 5.42]	7.55	0.37	7	6.76	<0.00001	0.174
	without	ref										
	with											
Preoperative Osserman stages		10	358	2040	Fixed	4.55 [3.23, 6.13]	30.74	0.0006	67	9.15	<0.00001	0.64
	I	ref										
	IIa + IIb + III + VI	2				2.57 [1.43,4.61]	0.01	0.94	0	3.16	0.002	
	IIb + III + VI	8				11.15 [6.88,18.08]	5.41	0.61	0	9.78	<0.00001	
	VI + V	1				1.80 [0.96, 3.36]	NA	NA	NA	1.84	0.002	
Postoperative lung infection		5	92	448	Fixed	6.49 [2.64, 15.98]	2.39	0.5	0	4.07	<0.00001	0.624
	without	ref										
	with											
Thymoma		5	234	1390	Fixed	2.96 [2.13, 4.13]	3.63	0.3	17	6.43	<0.00001	0.806
	no	ref										
	yes											
Preoperative dosage of pyridostigmine bromide		4	193	1103	Fixed	3.53 [2.47,5.03]	4.98	0.17	40	6.94	<0.00001	1
	≥240 mg											
	<240 mg	ref										
Preoperative serum AchR-Ab level (nm/L)		2	27	248	Fixed	8.74 [3.31, 23.08]	0.03	0.87	0	3.65	0.0003	1
	>100											
	≤100	ref										
Operation time		3	95	325	Random	1.18 [0.89, 1.57]	19.47	<0.00001	90	1.13	0.26	0.296
**Surgery-related risk factors**												
Intraoperative blood loss > 1000 ml		2	26	185	Fixed	15.03 [3.50, 64.50]	0.03	0.87	0	3.65	0.0003	1
	>1000 ml											
	≤1000 ml	ref										
WHO classification		2	25	190	Fixed	15.23 [4.63, 50.13]	0.22	0.64	0	4.48	<0.00001	1
	non-thymoma and non-invasive thymoma	ref										
	invasive thymoma											
Mode of operation		4	66	252	Fixed	5.88 [2.06,16.80]	2.09	0.55	0	3.3	0.001	0.089
	video-assisted thoracoscopy	ref										
	thoracotomy											
**Postoperative risk factors**												
Preoperative lung function		5	110	784	Fixed	5.71 [3.11, 10.48]	3.3	0.51	0	5.62	<0.00001	1
	normal	ref										
	abnormal											
Major postoperative complications		3	87	391	Fixed	33.78 [10.57,107.96]	1.59	0.45	0	5.94	<0.00001	1
	without	ref										
	with											
Disease duration before thymectomy		2	39	452	Fixed	5.45 [1.96, 15.14]	0.02	0.9	0	3.26	0.001	1
			1983	11,345								

### Risk factors

#### Risk factors related to the patients’ preoperative conditions (Fig. 2a)

##### Preoperative MC history

Twelve studies [8, 20–23, 27, 29, 31, 33–36] provided data on the history of MC and included 2094 patients. The results of the heterogeneity test did not reveal heterogeneity among the studies (I^2^ = 18%, *P* = 0.26); therefore, the fixed effect model was used for the meta-analysis. A preoperative history of MC was an independent risk factor for MCAT [OR = 4.13, 95% CI (3.08, 5.54), *P* < 0.00001].

**Fig. 2:**
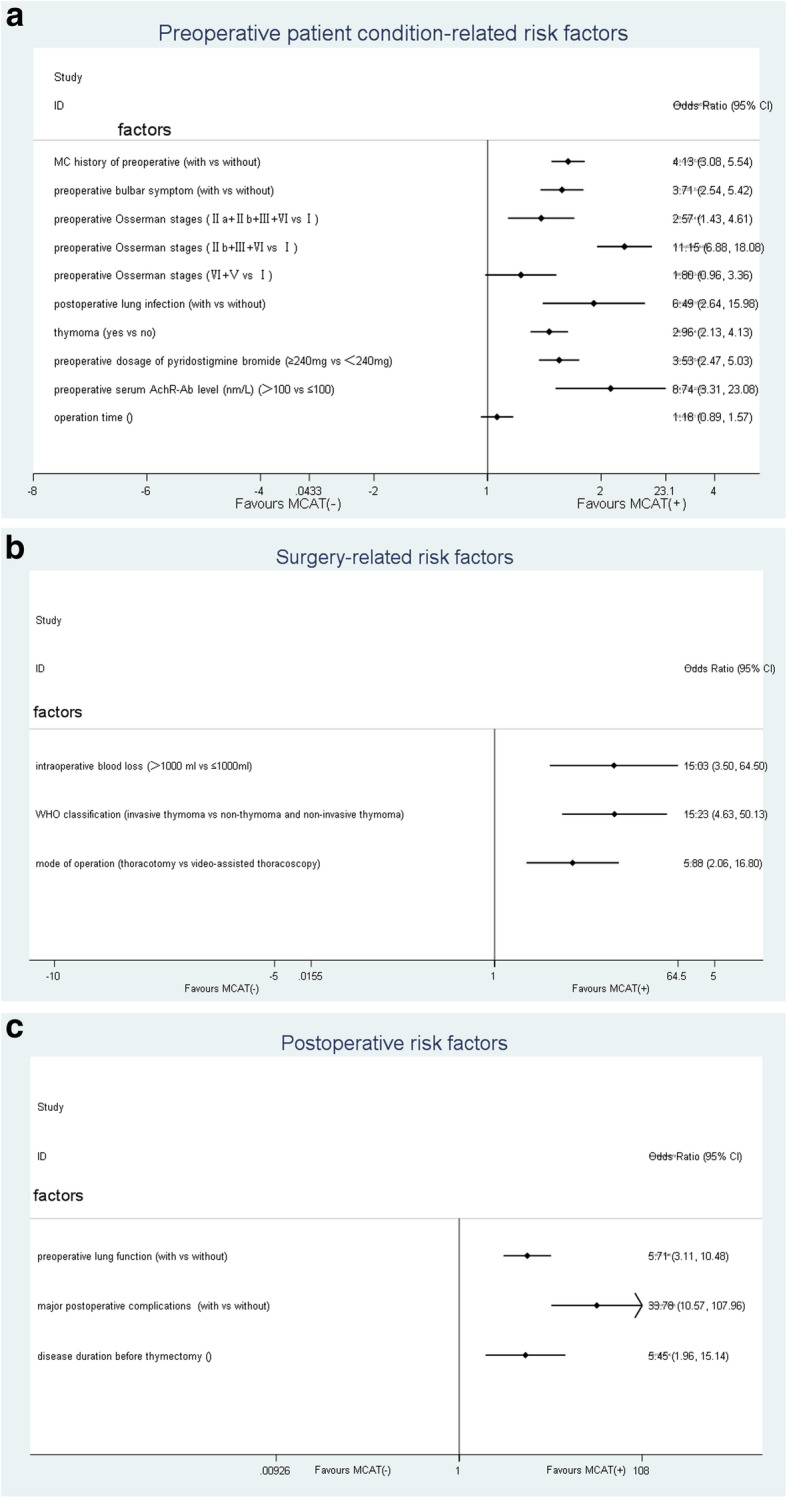
**a** Meta-analyses of the association between MCAT and risk factors related to the patients’ preoperative conditions. **b** Meta-analyses of the association between MCAT and surgery-related risk factors. **c** Meta-analyses of the association between MCAT and postoperative risk factors

##### Preoperative bulbar symptoms

Eight studies [7, 16–18, 21, 22, 27, 35] including 1443 patients reported the relationship between preoperative bulbar symptoms and MCAT. The results of the heterogeneity test did not reveal heterogeneity among the studies (I^2^ = 7%, *P* = 0.37), and thus the fixed effect model was used for the meta-analysis. Preoperative bulbar symptoms were an independent risk factor for MCAT [OR = 3.71, 95% CI (2.54, 5.42), *P* < 0.00001].

##### Preoperative Osserman stages

Ten studies [8, 19, 20, 22, 28–30, 32–34, 36] including 2040 patients provided the relationship between the preoperative Osserman classification and MCAT. The results of the heterogeneity test showed evidence of heterogeneity between studies (I^2^ = 67%, *P* = 0.0006). According to the preoperative Osserman stages, the patients were divided into three subgroups: IIa + IIb + III + VI, IIb + III + VI, and VI + V. The results of the heterogeneity test did not reveal any heterogeneity (I^2^ = 0, *P* > 0.1); therefore, the fixed effect model was used for the analysis. Notably, IIa + IIb + III + VI [OR = 2.57, 95% CI (1.43, 4.61), *P* = 0.002], IIb + III + VI [OR = 11.15, 95% CI (6.88, 18.08), *P* < 0.00001], and VI + V [OR = 1.80, 95% CI (0.96, 3.36), *P* = 0.07] were all statistically significant. Based on the analysis described above, preoperative Osserman stages were an independent risk factor for MCAT, and patients with preoperative type IIb + III + VI stages had the highest risk of MCAT.

##### Preoperative dosage of pyridostigmine bromide

Five studies [20, 22, 28, 29, 35] including 1103 patients provided data on the relationship between the preoperative pyridostigmine bromide dosage and MCAT. The results of the heterogeneity test showed evidence of heterogeneity between studies (I^2^ = 92%, *P* < 0.00001). After the sensitivity analysis, the study by Liu et al. [28] may have been the source of heterogeneity, and the heterogeneity test was repeated after excluding this study. No heterogeneity was observed between the studies (I^2^ = 40%, *P* = 0.17), which were analysed using a fixed effect model. Moreover, the dosage of pyridostigmine bromide before the operation was an independent risk factor for MCAT [OR = 3.53, 95% CI (2.47, 5.03), *P* < 0.00001].

##### Preoperative serum acetylcholine receptor antibody (AchR-Ab) level

Two studies [7, 36] reported the relationship between the preoperative AchR-Ab level and MCAT, including a total of 248 patients. The results of the heterogeneity test showed no evidence of heterogeneity between the studies (I^2^ = 0%, *P* = 0.89), and thus the fixed effect model was used for the meta-analysis. A preoperative AchR-Ab level > 100 nm/L was an independent risk factor for MCAT [OR = 8.74, 95% CI (3.31, 23.08), *P* < 0.00001].

##### Preoperative lung function

Five studies [17, 18, 22, 24, 28] including 784 patients reported the relationship between preoperative lung function and MCAT. The results of the heterogeneity test did not reveal heterogeneity among the studies (I^2^ = 0%, *P* = 0.51); therefore, the fixed effect model was used for the meta-analysis. The risk of MCAT in patients with abnormal preoperative lung function was 5.71 times higher than for patients with a normal preoperative pulmonary function. Preoperative pulmonary function was an independent risk factor for MCAT [OR = 5.71, 95% CI (3.11, 10.48), *P* < 0.00001].

##### Major preoperative complications

Three studies [15, 32, 34] including 391 patients reported the relationship between preoperative complications and MCAT. The results of the heterogeneity test did not detect heterogeneity between the studies (I^2^ = 0%, *P* = 0.45), and thus the fixed effect model was used for the meta-analysis. The risk of MCAT in patients with preoperative complications was 33.78 times higher than in patients without complications. Preoperative complications were independent risk factors for MCAT [OR = 33.78, 95% CI (10.57, 107.96), *P* < 0.00001].

##### Disease duration before thymectomy

Two studies [8, 18] including 452 patients reported the relationship between the preoperative disease duration before thymectomy and MCAT. The results of the heterogeneity test showed no heterogeneity between the studies (I^2^ = 0%, *P* = 0.90), and thus the fixed effect model was used for the meta-analysis. The preoperative course of disease was an independent risk factor for MCAT [OR = 5.45, 95% CI (1.96, 15.14), *P* = 0.001]. According to Kanai et al. [18], a disease duration< 3 months was a risk factor for MCAT [OR = 5.18, 95% CI (1.42, 18.8)]. Leuzzi et al. [8] identified a disease course of > 2 years as a risk factor for MCAT [OR = 5.94, 95% CI (1.12, 31.48)].

#### Surgery-related risk factors (Fig. 2b)

##### Operation time

Three studies [19, 27, 33] that included 325 patients provided data on the relationship between the operation time and MCAT. The results of the heterogeneity test showed substantial heterogeneity between studies (I^2^ = 90%, *P* < 0.00001). The random effect model was used for the analysis. The operation time was not an independent risk factor for MCAT [OR = 1.18, 95% CI (0.89, 1.57), *P* = 0.26].

##### Intraoperative blood loss> 1000 mL

Two studies [7, 19] reported the relationship between intraoperative blood loss and MCAT and included 185 patients. The results of the heterogeneity test showed no heterogeneity between the studies I^2^ = 0%, *P* = 0.87); therefore, the fixed effect model was used for the analysis. The risk of MCAT in patients with intraoperative blood loss > 1000 mL was 15.03 times greater than patients with an intraoperative blood loss ≤1000 mL. Intraoperative blood loss > 1000 mL was an independent risk factor for MCAT [OR = 15.03, 95% CI (3.50, 64.50), *P* = 0.0003].

##### Mode of operation

Four studies [15, 19, 25, 33] reported the relationship between the mode of operation and postoperative MC and included 252 patients. The results of the heterogeneity test did not identify heterogeneity among the studies (I^2^ = 0%, *P* = 0.55), and thus the fixed effect model was used for the meta-analysis. The risk of MCAT after thoracotomy was 5.88 times greater than after video-assisted thoracoscopy. The mode of operation was an independent risk factor for MCAT [OR = 5.88, 95% CI (2.06, 16.80), *P* = 0.0010].

#### Postoperative risk factors (Fig. 2c)

##### Postoperative lung infection

Five studies [8, 20, 22, 33, 34] including a total of 989 patients reported the relationship between postoperative lung infection and MCAT. The results of the heterogeneity test showed no evidence of heterogeneity between the studies (I^2^ = 0%, *P* = 0.45); therefore, the fixed effect model was used for the analysis. A postoperative lung infection was an independent risk factor for MCAT [OR = 4.43, 95% CI (2.38, 8.22), *P* < 0.00001].

##### Thymoma

Five studies [20, 21, 28, 29, 32] provided data on the relationship between thymoma and MCAT, including 1390 patients. The results of the heterogeneity test showed evidence of heterogeneity between studies (I^2^ = 79%, *P* = 0.0008). After the sensitivity analysis, the study by Chu et al. [32] may have been the source of heterogeneity, and the heterogeneity test was repeated after excluding this study. No heterogeneity was observed between the studies (I^2^ = 17%, *P* = 0.30), which were analysed using a fixed effect model. Thymoma was an independent risk factor for MCAT [OR = 2.96, 95% CI (2.13, 4.13), *P* < 0.00001].

##### World Health Organization (WHO) classification

Two studies [17, 19] reported the relationship between the postoperative thymic WHO classification and MCAT. The results of the heterogeneity test did not reveal heterogeneity between the studies (I^2^ = 0%, *P* = 0.64), and thus the fixed effect model was used for the meta-analysis. The risk of MC in patients with invasive thymoma was 15.23 times greater than in patients without thymoma and patients with noninvasive thymoma. The postoperative WHO classification was an independent risk factor for MCAT [OR = 15.23, 95% CI (4.63, 50.13), *P* < 0.00001].

## Discussion

### Main findings and interpretation of the results

The purpose of this meta-analysis is to provide evidence for the predictors of MCAT. An understanding of the risk factors for MCAT is helpful for doctors and patients to coordinate the optimal postoperative management strategy. Overall, 25 studies in our meta-analysis reported risk factors for MCAT based on an analysis of 11,345 patients, and the incidence of MCAT complications was 17.48%. The results of the meta-analysis identified the following factors related to the condition of patients before the operation as risk factors for MC: a history of preoperative MG, preoperative bulbar symptoms, preoperative Osserman stages, preoperative dosage of pyridostigmine bromide, preoperative serum acetylcholine receptor antibody (AchR-Ab) level (> 100 nm/L), preoperative lung function, major postoperative complications, and disease duration before thymectomy. Surgery-related risk factors were blood loss > 1000 mL and the mode of operation (thoracotomy). Postoperative risk factors were postoperative lung infection, thymoma, and the WHO classification.

A history of preoperative MG, preoperative bulbar symptoms, preoperative Osserman stages, and preoperative lung function were risk factors for postoperative MC, consistent with the results reported by AKaishi et al. [37] regarding the preoperative predictors of MCAT.

Preoperative Osserman stages reflect the severity of MG symptoms; type I is limited to extraocular muscles, type II and above include the involvement of other skeletal muscles, and patients with type IIb and above have symptoms of medulla oblongata muscle involvement, such as swallowing difficulty. Most of the studies [30, 32] reported that the preoperative Osserman stage was an independent factor affecting the occurrence of MCAT. Xue et al. [17] proposed that the WHO histological classification B2-B3 and Osserman stages IIa-IV independently predicted preoperative risks of a post-operative myasthenic crisis (POMC).

As shown in the study by Lucchi et al. [38], compared with type (I + IIa), type (IIb + III + IV) had a worse prognosis, which was related to the involvement of swallowing muscles before the operation. The potential explanations for the relationship between preoperative Osserman stages and the prognosis are listed below. First, type I and IIa symptoms are mainly concentrated in the ocular muscles and affect limb muscle strength, but do not involve the respiratory muscles. The symptoms associated with type IIb and above involve respiratory muscles, and patients with MG are prone to dyspnoea. Second, patients with type IIb and greater symptoms present with abnormal mastication and swallowing functions, which can easily cause coughing and choking while drinking water or the accumulation of saliva that is unable to swallowed and mistakenly passes into the airway, resulting in respiratory tract obstruction and crisis.

A history of preoperative MC and bulbar symptoms were identified as independent risk factors for developing POMC, which represented the severe status of MG. Additionally, in patients with systemic MG, the disease should be controlled to the greatest extent possible before the operation to reduce the preoperative stages, which is helpful to reduce the incidence of MCAT and modulate the long-term effect.

Few studies [7, 36] examined the correlation between the preoperative AchR-Ab titre and MCAT. The result of the meta-analysis showed that a high serum AchR-Ab level (AchR-Ab level > 100 nm/L) was related to the occurrence of MCAT.

The disease duration before thymectomy was an independent factor affecting the occurrence of MCAT in the present study. According to Leuzzi et al. [8], a symptom duration of > 2 years independently predicted POMC. A longer duration of MG was reported to contribute to the risk of a postoperative MC [8, 39]. A long disease duration is generally related to a poor treatment response [40], probably due to the cumulative damage at the neuromuscular junction.

In contrast, some reports [7, 27] revealed a tendency for a short disease duration to be related to the risk of a postoperative MC. Kanai et al. [18] identified a disease duration of < 3 months as a risk factor for POMC. The short course of the disease may reflect the rapid progression of MG or an insufficient response to treatment designed to inhibit the activity of the disease. The specific mechanism of MC and the course of the disease require further analysis.

Few reports have analysed the effect of MG associated with immune diseases on MCAT. Chen et al. [34] reported that concomitant immune disease (hyperthyroidism) was an important factor predicting the occurrence of postoperative MC. For patients with MG complicated with other autoimmune diseases, as indicated by a preoperative examination, close observation and early prevention measures should be implemented after the operation, which may reduce the possibility of postoperative MC to a certain extent. However, due to the inclusion of a limited number of cases, further studies are needed to determine and verify whether other autoimmune diseases are risk factors for long-term effects in later stages.

Thymectomy in patients with MG is divided into traditional thoracotomy and thoracoscopic surgery. According to our meta-analysis, thoracotomy was an independent risk factor for MCAT. This factor may be related to the trauma and bleeding associated with traditional thoracotomy, which easily causes postoperative pulmonary infection. Watanabe et al. [7] showed that intraoperative blood loss > 1000 mL was a prognostic factor for postoperative MC. The risk of MCAT in patients with intraoperative blood loss > 1000 mL was 18.52 times greater than that in patients with an intraoperative blood loss ≤1000 mL. The mechanism of intraoperative blood loss > 1000 mL affecting postoperative myasthenia crisis is unclear.

The dosage of pyridostigmine bromide before the operation is an independent risk factor for MCAT. An acetylcholinesterase inhibitor (AChEI) increases the functional concentration of acetylcholine by blocking acetylcholinesterase at the neuromuscular junction to improve muscle contraction, strength and function. The preoperative use of large doses of cholinesterase inhibitors will activate muscarinic receptors, accelerate the destruction of postsynaptic acetylcholine receptors (AChRs) at the neuromuscular junction, decrease the ability of patients to cough up sputum due to postoperative pain, cause excessive respiratory secretions after the operation, increase the risk of respiratory tract infection, and increase the risk of crisis.

The meta-analysis identified a postoperative pulmonary infection as the main risk factor for MCAT in patients with MG. Pulmonary infection increases respiratory secretions and reduces the area of the effective alveolar membrane, which leads to MCAT. However, a postoperative infection does not indicate that the MC lasts for a long time [41].

Pulmonary function tests directly evaluate the function of respiratory muscles and reflect the compliance of respiratory muscles. Prigent et al. [42] reported the therapeutic effect on ICU patients and the timing of endotracheal intubation and extubation in MG patients based on measurements of vital capacity (VC).

Preoperative pulmonary ventilation function plays an important role in predicting MCAT. Pulmonary function tests directly evaluate the function and reflect the compliance of respiratory muscles. Weakness of these muscles can be evaluated by the forced vital capacity (FVC), and these measurements are useful to detect respiratory muscle failure in MG [42]. As shown in the study by Loach et al. [43], patients with MG presenting a preoperative VC > 2 L were less likely to experience a postoperative crisis. Choi et al. [26] showed that the optimal cut-off point of preoperative pulmonary function to determine the risk of MCAT was 65% of the mean FVC (mFVC)/peak FVC (pFVC), indicating that patients with a significantly reduced mFVC/pFVC value may not be candidates for thymectomy. Thymectomy is not suitable for patients with MG presenting poor pulmonary function but may be considered after stabilization, and these patients should be closely monitored during the postoperative period.

The relationship between thymoma and postoperative crisis has been controversial [44, 45]. The meta-analysis identified thymoma as an independent risk factor for MCAT, and pathological types (invasive thymoma, B2-B3) were independent risk factors for MCAT. A potential explanation for this finding is that invasive thymoma is generally so invasive that it is difficult to remove during the operation, and thus the tumour (particularly type B thymoma) has a high probability of metastasis.

The meta-analysis did not identify the operation time as an independent risk factor for MCAT. Nevertheless, we are unable to exclude this variable as a potential risk factor, as some studies [19, 27, 33] have reported a significant association of the operation time with MCAT. The results of this study exhibited substantial heterogeneity. Because the original data were unable to be obtained, a subgroup analysis was not conducted to explore the source of its heterogeneity.

### Implications from this meta-analysis

#### Limitations

Our research has several limitations that should be explained. First, because the studies we included are retrospective cohort studies and retrospective case-control studies and the related risk factors for MCAT are diverse and complex, these factors are affected by selection bias to some extent. Randomized controlled trials are difficult to perform, and selection bias caused by researchers and patients in clinical studies is inevitable. Second, some of the included research samples are relatively small, which once again indicates the need for further high-quality, large cohort studies in the future. In addition, the definition of MCAT is not uniform, the follow-up time is not consistent, and the outcome may thus be affected to some extent. Finally, individual studies lack detailed data, and consequently, no further subgroup analyses were able to be conducted to explore the sources of heterogeneity.

## Conclusions

This study combines the most recent evidence of risk factors for MCAT in patients with MG. The results of this meta-analysis confirm that the pathogenesis of MCAT is unclear and many risk factors for MCAT exist. The independent risk factors for MCAT were a preoperative history of MC, preoperative bulbar symptoms, preoperative MG Osserman stages, preoperative dosage of pyridostigmine bromide, preoperative serum AchR-Ab level (> 100 nm/L), lung function, major postoperative complications, disease duration before thymectomy, blood loss> 1000 mL, thoracotomy, postoperative lung infection, thymoma, and WHO classification.

## Supplementary information

**Additional file 1: Table S1.** NOS criteria were used to evaluate the quality of the 25 included studies.

## Data Availability

The datasets generated and analysed are available from the corresponding author on reasonable request.

## Abbreviations

MG: Myasthenia gravis
MCAT: myasthenic crisis after thymectomy
AchR-Ab: anti-acetylcholine receptor antibody
MC: MG crisis
NOS: Newcastle-Ottawa Scale
MD: Mean difference
WHO: World Health Organization
VC: Vital capacity
POA: preoperative anxiety
