# Supplementary material for: Assessment of the risks of a myasthenic crisis after thymectomy in patients with myasthenia gravis: a systematic review and meta-analysis of 25 studies
Source: J Cardiothorac Surg. 2020 Sep 29;15:270. doi: 10.1186/s13019-020-01320-x (PMC7526111; doi:10.1186/s13019-020-01320-x)
Supplement: Supplementary file 1 — Additional file 1: Table S1. NOS criteria were used to evaluate the quality of the 25 included studies. [file 13019_2020_1320_MOESM1_ESM.doc]

**Table 1 in the supplement:** NOS criteria were used to evaluate the quality of the 25 included studies.

| **Study and year** | **Selection** | **Comparability** | **Exposure**/Outcome | NOS scores |
| --- | --- | --- | --- | --- |
| Kato, 2019 | ☆☆☆ | ☆☆ | ☆☆☆ | 8 |
| Li, 2016 | ☆☆☆ | ☆☆ | ☆☆☆ | 8 |
| Qian, 2016 | ☆☆☆ | ☆☆ | ☆☆☆ | 8 |
| Li, 2017 | ☆☆☆ | ☆☆ | ☆☆ | 7 |
| Liu, 2014 | ☆☆ | ☆☆ | ☆☆ | 6 |
| Niu, 2013 | ☆☆ | ☆☆ | ☆☆ | 6 |
| Ma, 2011 | ☆☆ | ☆☆ | ☆☆ | 6 |
| Zhang, 2015 | ☆☆ | ☆☆ | ☆☆ | 6 |
| Wang, 2006 | ☆ | ☆☆ | ☆☆ | 5 |
| Chen, 2007 | ☆☆☆ | ☆☆ | ☆☆☆ | 8 |
| Ge, 2019 | ☆☆☆☆ | ☆ | ☆ | 6 |
| Li, 2014 | ☆ | ☆☆ | ☆☆ | 5 |
| xue, 2017 | ☆☆☆ | ☆☆ | ☆☆☆ | 8 |
| Kanai, 2017 | ☆☆☆☆ | ☆☆ | ☆☆☆ | 9 |
| Zou, 2016 | ☆☆☆☆ | ☆ | ☆ | 6 |
| Ando, 2015 | ☆☆ | ☆☆ | ☆☆☆ | 7 |
| Lee, 2015 | ☆☆☆ | ☆☆ | ☆☆☆ | 8 |
| Liu, 2006 | ☆☆☆☆ | ☆☆ | ☆☆☆ | 9 |
| Li, 2018 | ☆☆☆☆ | ☆☆ | ☆☆☆ | 9 |
| Yu, 2014 | ☆☆☆☆ | ☆ | ☆☆☆ | 8 |
| Choi, 2014 | ☆☆ | ☆ | ☆☆☆ | 6 |
| Leuzzi, 2014 | ☆☆☆☆ | ☆☆ | ☆☆☆ | 9 |
| Chu, 2011 | ☆☆ | ☆ | ☆☆ | 5 |
| Nam, 2011 | ☆☆☆☆ | ☆☆ | ☆☆☆ | 9 |
| Watanabe, 2004 | ☆☆☆☆ | ☆ | ☆ | 6 |
